# AII amacrine cells in the primate fovea contribute to photopic vision

**DOI:** 10.1038/s41598-018-34621-2

**Published:** 2018-11-06

**Authors:** Enrica Strettoi, Rania A. Masri, Ulrike Grünert

**Affiliations:** 1 CNR Neuroscience Institute, Pisa, 56124 Italy; 2 Save Sight Institute, Discipline of Clinical Ophthalmology, The University of Sydney, Sydney, NSW 2000 Australia; 3 Australian Research Council Centre of Excellence for Integrative Brain Function, The University of Sydney, Sydney, NSW 2000 Australia; 4 Faculty of Medicine and Health, The University of Sydney, Sydney, Australia

## Abstract

The AII amacrine cell is known as a key interneuron in the scotopic (night-vision) pathway in the retina. Under scotopic conditions, rod signals are transmitted via rod bipolar cells to AII amacrine cells, which split the rod signal into the OFF (via glycinergic synapses) and the ON pathway (via gap junctions). But the AII amacrine cell also has a “day job”: at high light levels when cones are active, AII connections with ON cone bipolar cells provide crossover inhibition to extend the response range of OFF cone bipolar cells. The question whether AII cells contribute to crossover inhibition in primate fovea (where rods and rod bipolar cells are rare or absent) has not been answered. Here, immunohistochemistry and three-dimensional reconstruction show that calretinin positive cells in the fovea of macaque monkeys and humans have AII morphology and connect to cone bipolar cells. The pattern of AII connections to cone bipolar cells is quantitatively similar to that of AII cells outside the fovea. Our results support the view that in mammalian retina AII cells first evolved to serve cone circuits, then later were co-opted to process scotopic signals subsequent to the evolution of rod bipolar cells.

## Introduction

Night-time (scotopic) vision is mediated by the well-described classical rod pathway involving rods, rod bipolar and AII amacrine cells [reviewed by^1,2^]. Rods contact rod bipolar cells, which depolarize in response to light. Rod bipolar cells transfer the rod signal to AII cells, which in turn make sign-conserving electrical synapses (gap junctions) with ON cone bipolar cells, and sign-inverting glycinergic synapses with OFF cone bipolar cells. These cone bipolar cells synapse with ganglion cells thus transferring the rod signal into the cone pathways^3–6^.

More recently, AII amacrine cells were shown to contribute to daylight (photopic) vision [reviewed by^7,8^]. In daylight, cone signals can reach AII amacrine cells via gap junctions with ON cone bipolar cells. The ON pathway can then inhibit the OFF pathway via the glycinergic synapses between AII amacrine and OFF cone bipolar cells and OFF ganglion cells. This arrangement underlines cross-over inhibition, which extends the operating range of OFF ganglion cells in photopic conditions^9,10^.

A unique feature in the retina of primates including humans is the fovea: a morphological specialization in the central retina which is responsible for high acuity vision. The centre of the fovea (the *foveola*) is characterized by a high cone density and a rod free zone^11–14^. The first rod outer segments in humans and macaque appear at eccentricities of about 0.3 to 0.5 degrees, then rod density rises rapidly and exceeds cone density for eccentricities above 500 µm (~1.8 deg) in human retina and 400 µm (~2 deg) in macaque retina^12,13^.

The densities of rod bipolar^15,16^ and AII amacrine cells across the retina are well studied in macaque monkeys and it has been shown that in central retina the density of AII amacrine cells sets the limit (“bottleneck”) for scotopic spatial acuity^17,18^. AII amacrine cells in macaque and human are immunoreactive to antibodies against the calcium binding protein calretinin^17–21^. However, it has also been proposed that in the fovea antibodies against calretinin label a different type of glycinergic amacrine cell and that AII cells are absent from the fovea^19^. The present study addresses the questions (1) whether AII amacrine cells are present in the foveal centre, where rods and rod bipolar cells are vanishingly sparse, (2) how the architecture and fundamental connectivity of foveal AII amacrine cells are influenced by the absence of rod bipolar cells.

## Results

### Definitions

Following the terminology given by Polyak^22^ (see also refs^23,24^) the term “central retina” (or area centralis) refers to the central 10° of visual angle and comprises four concentric zones (foveola, fovea, parafovea and perifovea). In human fovea one degree of visual angle is equivalent to 0.285 mm; in macaque fovea one degree is equivalent to about 0.2 mm. Thus, the central area in human retina has a diameter of about 3 mm in human and in macaque the diameter is about 2 mm. The macula lutea (or macula) contains the yellow pigment, it is 4 to 6° in diameter and thus slightly smaller than the area centralis. The most central zone of the central retina, the foveola (or fovea centralis) contains the highest density of cones and is characterized by the absence of blood vessels and all inner retinal layers. The foveola represents approximately the central 1.3° of visual angle and has a diameter of 250 µm to 350 µm. The term fovea refers to about 5.5° of visual angle, therefore, the diameter of the fovea is about 1.6 mm in human and 1.1 mm in macaque. The fovea contains all retinal layers including a thick ganglion cell layer (up to eight cells deep in human and up to six cells deep in macaque). In the present study, we use the term fovea to refer to eccentricities below 0.6 mm (~3 deg) in macaque and below 0.8 mm (~3 deg) in human retina because no significant connections with rods and rod bipolar cells are to be expected at eccentricities below 3 degrees.

### The majority of calretinin positive amacrine cells in the fovea are All cells

**Figure 1:**
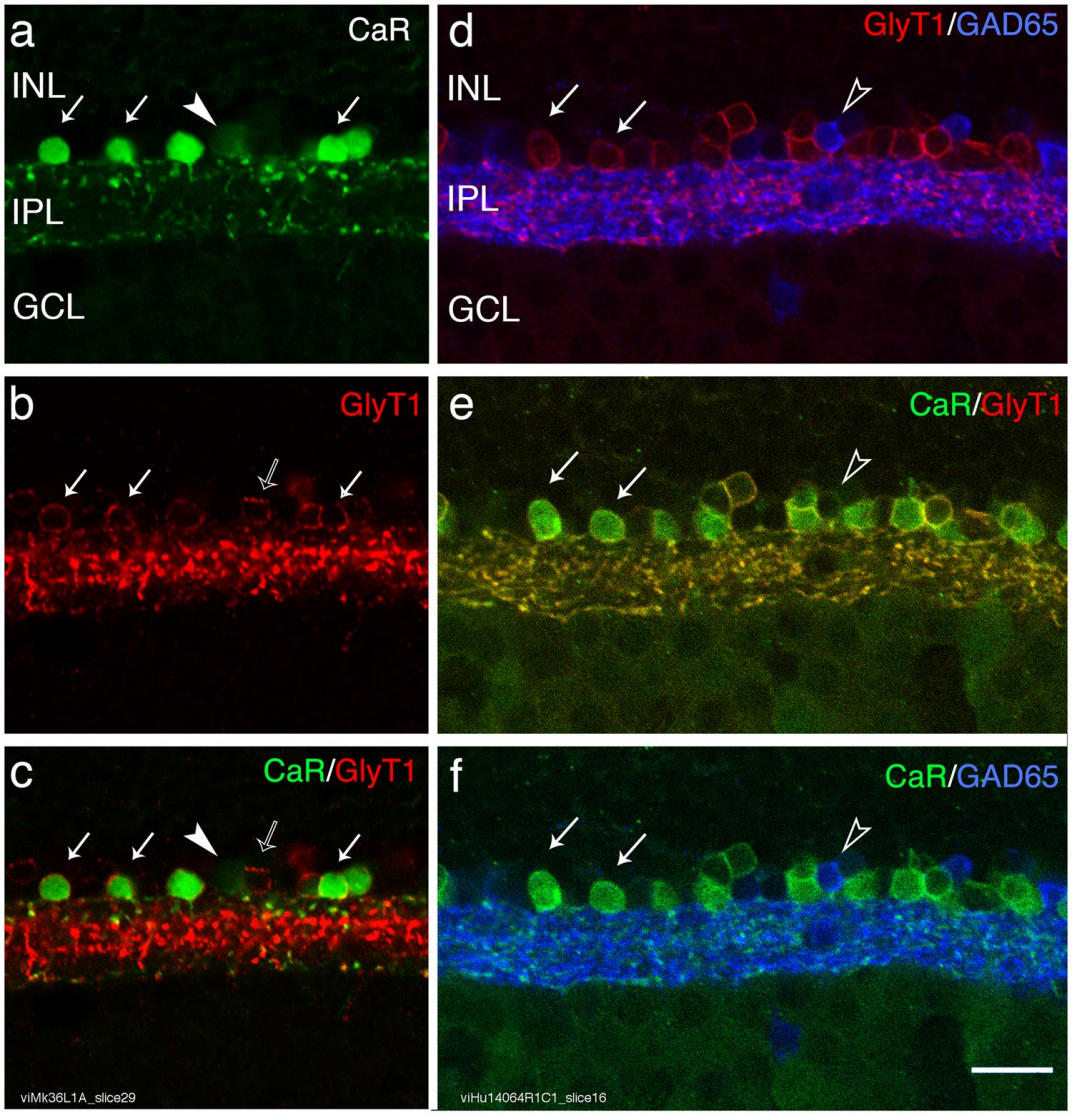
The majority of calretinin positive amacrine cells is glycinergic. (**a–c**) Macaque retina: Confocal images of a vibratome section through central retina processed with antibodies against calretinin (CaR, green) and antibodies against glycine transporter 1 (GlyT1, red). The white arrows indicate double labelled cells, the arrowhead points to a calretinin positive/GlyT1 negative cell and the open arrow point to a GlyT1 positive/CaR negative cell. (**d–f**) Human retina: Confocal images of a vibratome section through central retina processed with antibodies against calretinin (CaR, green), antibodies against glycine transporter 1 (GlyT1, red), and antibodies against glutamic acid decarboxylase (GAD, blue). The open arrowheads indicate a GAD positive/CaR positive cell. INL, inner nuclear layer; IPL, inner plexiform layer; GCL, ganglion cell layer. Scale bar shown in f = 20 µm (valid for **a–f**).

Using double and triple labelling with antibodies against calretinin, glycine transporter 1 (GlyT1) and glutamic acid decarboxylase (GAD) we found that in both macaque (Fig. 1a–c) and human (Fig. 1d–f) retina nearly all calretinin positive cells express GlyT1 immunoreactivity and only a small population of calretinin positive cells are immunoreactive to GAD antibodies. This finding is consistent with previous studies^19–21,25^. The calretinin positive/GlyT1 positive cells had the morphology of AII cells (Fig. 1a–c, and see below), calretinin-positive processes were arranged in two bands in the inner plexiform layer, with the outermost tier composed of large, globular varicosities, known as lobular appendages. We compared the proportion of calretinin positive/GlyT1 positive cells (presumed AII cells) among the population of calretinin cells in the fovea to that outside the fovea (0.6–1.5 mm eccentricity). In human, the proportion of AII cells among calretinin-positive cells (65.5% ± 13.9 SD, n = 460 AII cells) was only marginally lower in the fovea than outside the fovea (71.9% ± 5.6%, n = 310 AII cells, p = 0.07, χ2 test). In macaque, AII cells made up the majority of calretinin-positive cells in the fovea (71.8% ± 25.1% SD, n = 54) but their proportion was significantly lower than that outside the fovea (95.8% ± 4.6, n = 327 AII cells, p < 0.01, χ2 test).

Similar results were obtained from sections that were labelled with antibodies against calretinin and antibodies against GAD (Fig. 1d–f). Assuming that all calretinin positive but GAD negative cells are AII cells, we found that in human 91.5% ± 7.5% SD of the calretinin cells in foveal retina were AII cells (n = 1112), versus 96.1% ± 2.1 outside the fovea (n = 643 cells, p = 0.01, χ2 test). In macaque 86.2% ± 7.9% of the foveal calretinin cells were AII cells (n = 107) versus 94.1% ± 3.8% outside the fovea (n = 357, p < 0.01, χ2 test). The lower percentage of presumed AII cells obtained in human retina when defined as calretinin positive/GlyT1 positive cells (range 72–95%, see above) compared to calretinin positive/GAD negative cells (range 86–96%) is likely due to an underestimate of the calretinin positive/GlyT1 double labelled cells. In one of the sections through human retina we used a calretinin antibody made in mouse whereas in all other sections we used a calretinin antibody made in goat. The antibody made in mouse consistently yielded less strongly labelled cells. This discrepancy notwithstanding, our results show that AII cells form the majority of calretinin-positive amacrine cells in the fovea, but their contribution to the calretinin positive amacrine cells is somewhat lower than that outside of the fovea.

### Rods, rod bipolar, and AII amacrine cells in central retina

**Figure 2:**
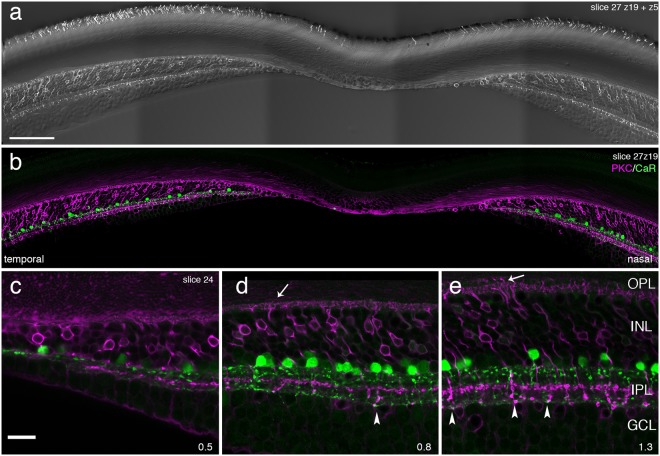
Rod pathway neurones in macaque fovea. Confocal images of vibratome sections through the fovea that were processed with antibodies against rhodopsin to reveal rods, antibodies against protein kinase C (PKC) to reveal rod bipolar cells and antibodies against calretinin (CaR). (**a**) Photomontage of two confocal planes (z5) to show rod labelling and (z19) to show rod bipolar labelling. (**b**) Same section as in a (z19) showing PKC labelled bipolar cells (magenta) and calretinin labelled amacrine cells (green). (**c–e**) The nasal area of a different section from the same preparation is shown. The arrowheads point to rod bipolar axon terminals, the arrows point to rod bipolar dendrites. OPL, outer plexiform layer; INL, inner nuclear layer; IPL, inner plexiform layer; GCL, ganglion cell layer. Scale bar shown in a = 100 µm (valid for **a,b**); scale bar shown in c = 20 µm (valid for **c–e**).

Rod bipolar cells in mammalian including primate retinas are immunoreactive to antibodies against protein kinase Cα (PKC)^15,26^. In order to compare the distribution of the three major players in the rod pathway (rods, rod bipolar cells, and AII amacrine cells) in the same preparation, we processed vibratome sections of macaque retina with antibodies against rhodopsin, PKC, and/or calretinin (Fig. 2). Consistent with previous studies^12^, in macaque the first scattered rod outer segments were detected within 40 µm of the foveal centre and their density increases rapidly with increasing eccentricity, exceeding the density of cones at eccentricities above 400 µm (~2 deg.). It also has been shown previously that in macaque antibodies against PKC not only label rod bipolar cells but also a type of diffuse bipolar cell (DB4)^16^. The two cell types can be distinguished by their dendritic morphology and their axonal stratification at two different depths in the inner plexiform layer. Figure 2c–e shows DB4 dendrites contacting cone pedicles as well as rod bipolar dendrites passing cone pedicles to reach rod spherules (arrows). The rod bipolar axon terminals end close to the ganglion cell layer (arrowheads), whereas the axons of DB4 cells form a band near the centre of the inner plexiform layer. In macaque, the first rod bipolar cells (i.e. the first contacts between rods and rod bipolar cells) were detected at an eccentricity of approximately 600 µm (Fig. 2d). Given the appearance of rod outer segments at around 100 µm, our value of 600 µm is consistent with the reported length of rod Henle fibres (between 300 and 400 µm; not corrected for shrinkage)^27^ in Golgi-impregnated macaque retina at this eccentricity.

**Figure 3:**
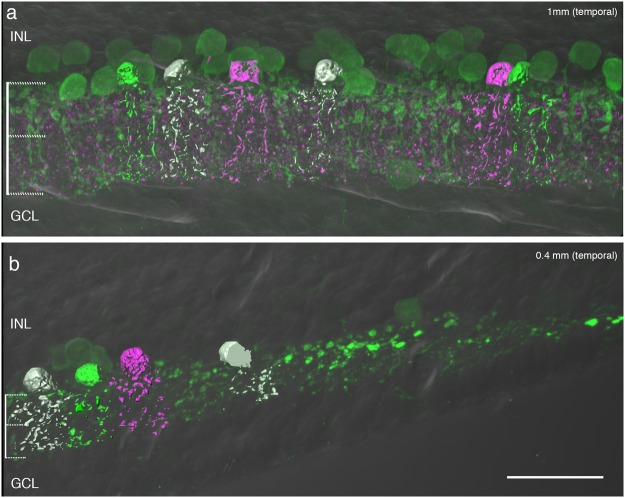
Reconstructions of AII amacrine cells in macaque retina. Three-dimensional views of a vertical vibratome section at (**a)** 1 mm and (**b)** 0.4 mm eccentricity. The section was processed with antibodies against calretinin (green) and calretinin positive cells were reconstructed. The OFF and ON sublamina of the inner plexiform layer are indicated on the left of the image. INL, inner nuclear layer; GCL, ganglion cell layer. Scale bar shown in b = 25 µm (valid for a and b).

Previous studies of macaque retina have shown that calretinin positive cells are present as close as 100 µm to the centre of the fovea^18,19^. These cells include a small proportion of GABAergic wide field amacrine cells but, as we have shown above, most calretinin-positive cells are glycinergic. To further support our conclusion that calretinin-positive cells in the fovea are AII amacrine cells, we reconstructed these cells from vibratome sections through the fovea of a macaque retina and compared their morphology within the fovea and at about 1 mm eccentricity. We found that most cells had AII morphology (Fig. 3a,b), with the first AII cells appearing at about 140 µm (0.7 deg) of the foveal centre. We compared the number of rod bipolar and presumed AII cells at three retinal eccentricities using morphological criteria (see below) in a 100 µm thick section through the centre of the fovea. At eccentricities between 50 and 250 µm a total of ten AII cells but no rod bipolar cells were found. At eccentricities between 300 and 400 µm there were 53 rod bipolar and 17 AII cells, and at eccentricities between 900 and 1000 µm there were 89 rod bipolar and 33 AII cells. Thus, the ratio between rod bipolar to AII cells increases from 0 in the fovea to about 3 at eccentricities between 300 to 1000 µm.

**Figure 4:**
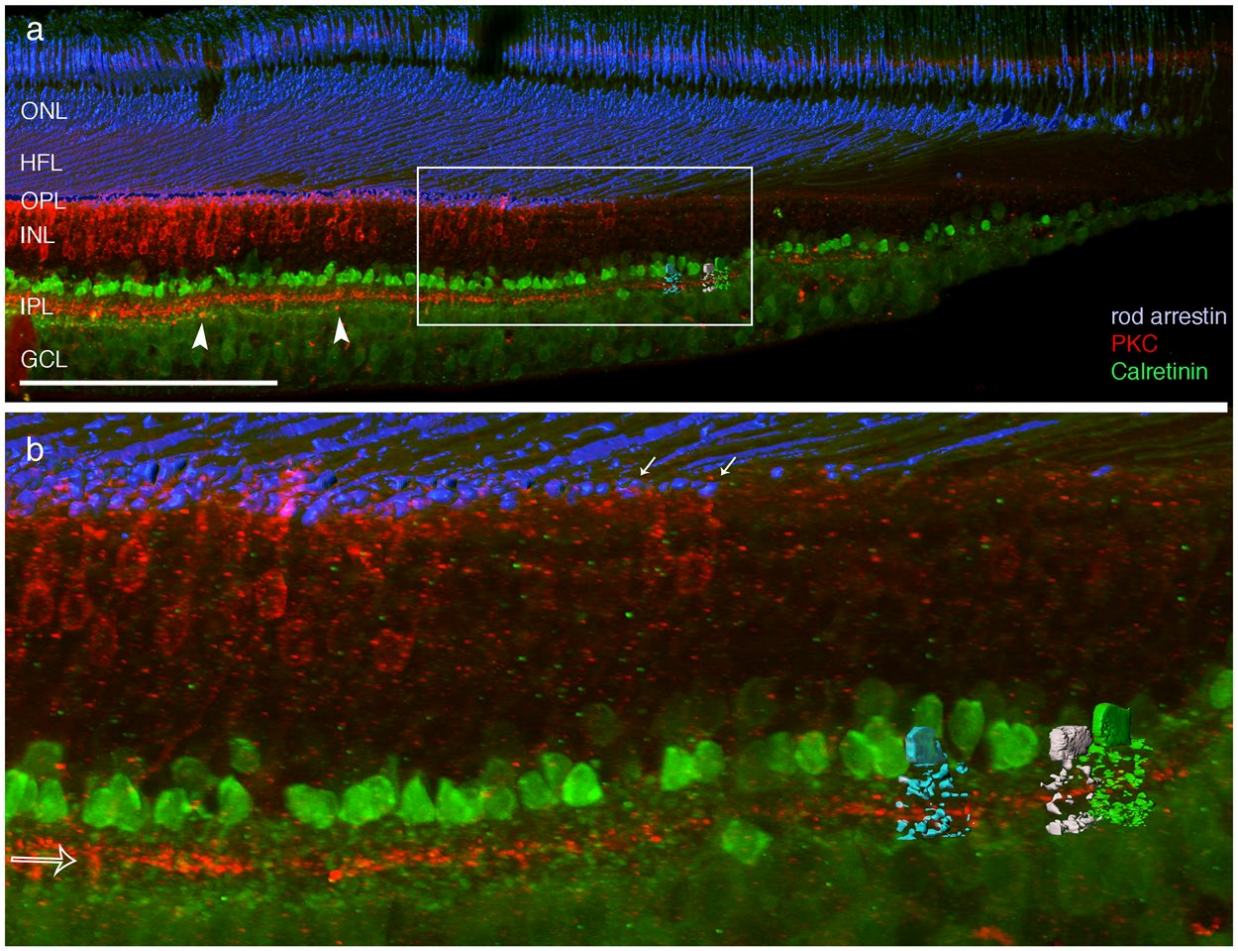
Rod pathway neurones in human fovea. (**a,b**) Confocal images of a vibratome section through the fovea that was processed with antibodies against rod arrestin to reveal rods (blue), antibodies against protein kinase C (PKC, red) to reveal rod bipolar cells and antibodies against calretinin (green). Rods are shown in 3d reconstruction. The arrowheads point to PKC labelled rod bipolar axon terminals. (**b**) Shows the region outlined in a. Three calretinin positive cells are reconstructed and have AII morphology. The open arrow points to the PKC labelled band formed by DB4 axons. The white arrows point to the first rod spherules appearing in the fovea. ONL, outer nuclear layer; HFL, Henle fibre layer, OPL, outer plexiform layer; INL, inner nuclear layer; IPL, inner plexiform layer; GCL, ganglion cell layer. Scale bar shown in a = 200 µm.

In human retina, we obtained similar qualitative results (Fig. 4). Rods were identified using antibodies against rod arrestin (s-antigen) which label the entire rod including its Henle fibre. Consistent with previous studies^13,14^, the first rod outer segments were found at about 70 µm distance from the foveal centre and the first rod spherules were found at about 600 µm from the fovea. Thus, the rod Henle fibres at this eccentricity are assumed to have a length of about 700 µm. Consistent with the first appearance of rod spherules, the somas of PKC labelled rod bipolar somas were first seen at about 600 µm and their axon terminals were first seen at about 800 µm eccentricity. The somas of PKC labelled DB4 cells were only very weakly labelled in human retina but their axon terminals are clearly visible near the centre of the inner plexiform layer at eccentricities from about 240 µm (open arrow in Fig. 4b). In contrast, rod bipolar axons terminate close to the ganglion cell layer and are first detected at eccentricities above 800 µm (Fig. 4a). Calretinin positive cells in human were found from about 200 µm (0.7 deg) of the foveal centre (Fig. 4a) and most of these cells had AII morphology (Fig. 4b). These results show that AII amacrine cells are present in a region of the fovea where rods and rod bipolar cells are absent.

### Morphology of AII cells in the fovea

Previous studies have shown that the morphology of calretinin positive cells in central and peripheral area of macaque retina resembles that of Golgi-impregnated^17^ and intracellularly injected AII cells^18^. Using an unbiased system to generate 3d profiles, we reconstructed calretinin positive cells of macaque retina within the fovea (n = 58 cells) and outside the fovea (n = 57 cells) from stacks of consecutive confocal images. We found that AII cells inside and outside the fovea have comparable morphology to that described in these previous studies (Fig. 3a,b). As in other mammals, individual cells are characterized by dendrites clearly clustered in the OFF sublamina and the ON sublamina of the inner plexiform layer. The outer tier is composed of large globular varicosities and the inner tier displays longer and radially oriented processes. Individual AII cells are smaller in fovea than in periphery: their dendrites in the ON sublamina are finer, and the bistratified morphology is less evident as result of distortion of the retinal layers by the foveal slope.

Reconstructions of calretinin immunoreactive cells in human fovea (Fig. 4a,b) revealed similar morphology, supporting the idea that antibodies against calretinin are markers for AII amacrine cells in human retina^20,21^. In the present study, calretinin positive cells with distinct AII morphology were obtained in only one preparation of human retina, which was fixed within 3.5 hours after death. The two other human retinas we studied were fixed more than 4 hours after death. Although these retinas had an excellent overall morphology and both rods and rod bipolar cells looked normal, the dendritic processes of calretinin positive amacrine cells were not labelled sufficiently. Thus, our quantitative study of synaptic connectivity of AII cells was restricted to sections from macaque retina. The morphological data and double labelling results described above from human retina nevertheless confirm that AII cells are present in both macaque and human fovea, giving a common basis for connectivity of AII cells to cone circuits in the fovea.

### Connectivity of AII amacrine cells to foveal cone circuits

If AII amacrine cells participate in cone circuits in the fovea, then they should show a similar pattern of connectivity with cone bipolar cells as known from peripheral retina. To answer this question, we labelled vibratome sections of macaque monkey retina with antibodies against calretinin and antibodies against CtBP2 to identify synaptic ribbons^28^ or with antibodies against connexin 36 to identify gap junctions^29,30^. Calretinin positive AII cells were reconstructed as described above and the density of synaptic connections was estimated from the number of immunoreactive puncta located close to the surface of the dendrites.

**Figure 5:**
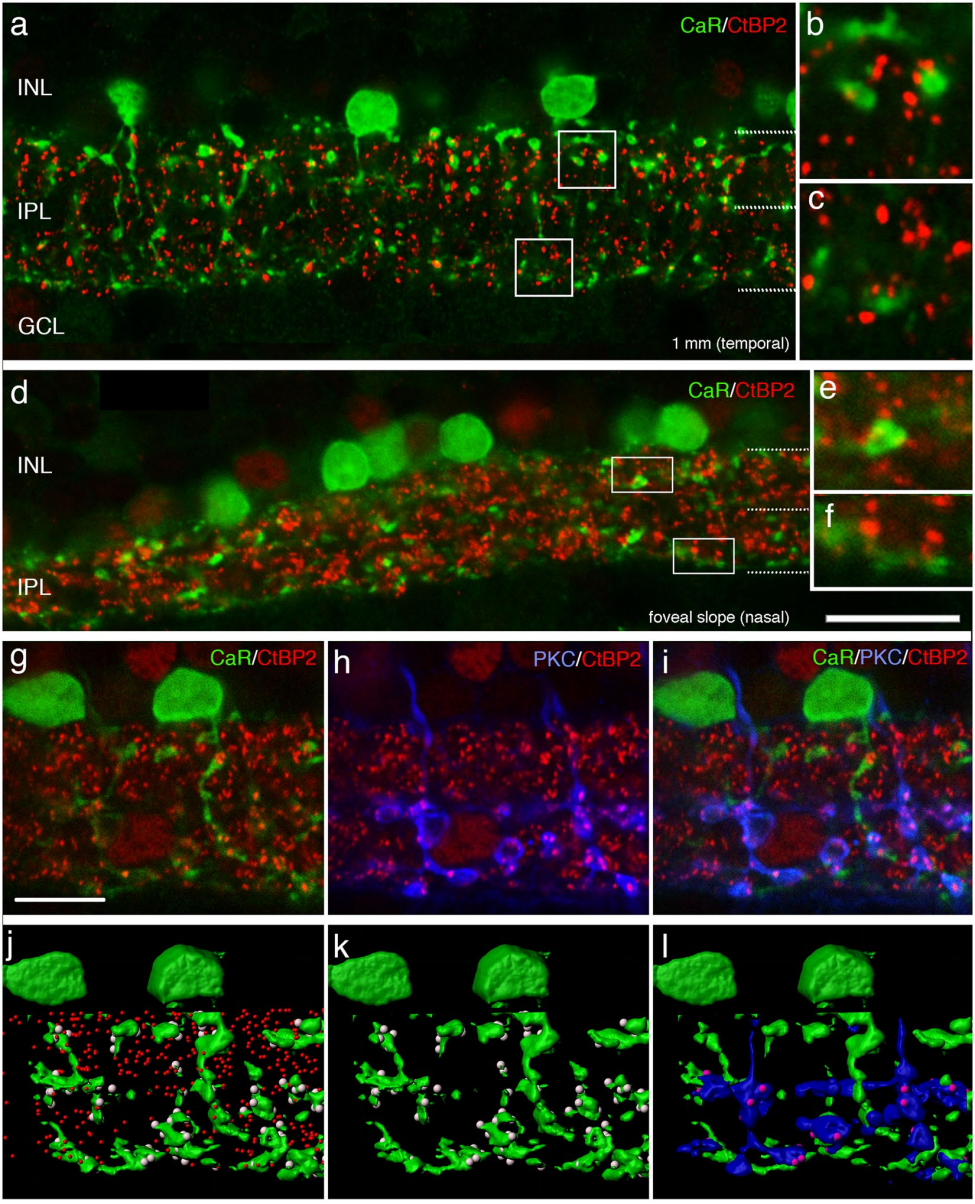
Bipolar connectivity of AII amacrine cells. Confocal images of a vertical vibratome section through macaque retina processed with antibodies against calretinin (CaR, green and the ribbon marker CtBP2, red). (**a**) Central retina, the boxes indicate the regions shown in b and c. Synaptic ribbons are closely associated with the dendrites of AII cells in both the ON and OFF sublamina of the inner plexiform layer. (**d**) Foveal slope, the boxes indicate the regions shown in e and f. (**g–i**) Confocal images of a section that was triple labelled with antibodies against calretinin (green), protein kinase C (blue) and antibodies against CtBP2 (red). (**j–l**) Three dimensional reconstructions of the AII and rod bipolar cells shown in g to i. (**j**) Shows the AII cells together with all synaptic ribbons in that area (red). Synaptic ribbons within 0.3 µm are shown in grey. (**k**) The same cells as in j together with associated synaptic ribbons; (**l**) the AII cells are shown together with rod bipolar axons (blue) and synaptic ribbons associated with both cell types (magenta). INL, inner nuclear layer; IPL, inner plexiform layer; GCL, ganglion cell layer. Scale bar shown in d = 20 µm (valid for a and d). Scale bar shown in g = 10 µm (applies to g to i).

We reconstructed 42 calretinin positive AII amacrine cells in the fovea and 22 calretinin positive AII cells outside the fovea and counted the CtBP2 positive puncta within a distance of 0.3 µm from the surface of the lobular appendages and arboreal dendrites for each cell. The CtBP2 immunoreactive puncta located within this distance are assumed to represent ribbons located within bipolar cells that are either pre- or postsynaptic to AII amacrine cells. As shown in Fig. 5, CtBP2 positive puncta associated with AII dendrites were found both in the OFF and in the ON sublamina. Previous studies in various mammals including macaque have shown that in the OFF sublamina, AII cells make output onto OFF cone bipolar cells but they also receive input from OFF cone bipolar cells^5,17,31,32^. Thus, we assume ribbons located close to the lobular appendages of an AII cell may include output synapses from AII cells onto cone bipolar axons as well as input synapses from cone bipolar cells onto AII amacrine cells.

Ribbons located close to AII cells in the ON sublamina were assumed to represent mostly input synapses from rod bipolar cells to AII cells (see below). These observations, however, leave open the possibility that some of the ribbons belong to cone bipolar cells stratifying in the vicinity of rod bipolar cells^33^. Thus, we used a triple labelled preparation to address this question. AII amacrine cells were labelled with antibodies against calretinin, synaptic ribbons were labelled with antibodies against CtBP2, and rod bipolar cells were labelled with antibodies against PKC (Fig. 5g–i). We reconstructed two AII cells and two rod bipolar axon terminals and determined the number of synaptic ribbons associated with the two cell types. In the reconstructions shown in Fig. 5j,k a total of 28 puncta were located within 0.3 µm of the lobular appendages of the two AII cells, and 87 puncta were located within 0.3 µm of the AII arboreal dendrites in the ON sublamina. In the rod bipolar cells a total of 95 puncta were co-localised with the axon terminals. A total of 67 puncta were associated with both the AII and the rod bipolar cells (magenta dots in Fig. 5l). Thus, we conclude that the majority (67/87 or 77%) of the puncta associated with AII cells in the ON sublamina represent rod bipolar input to the AII cell.

**Figure 6:**
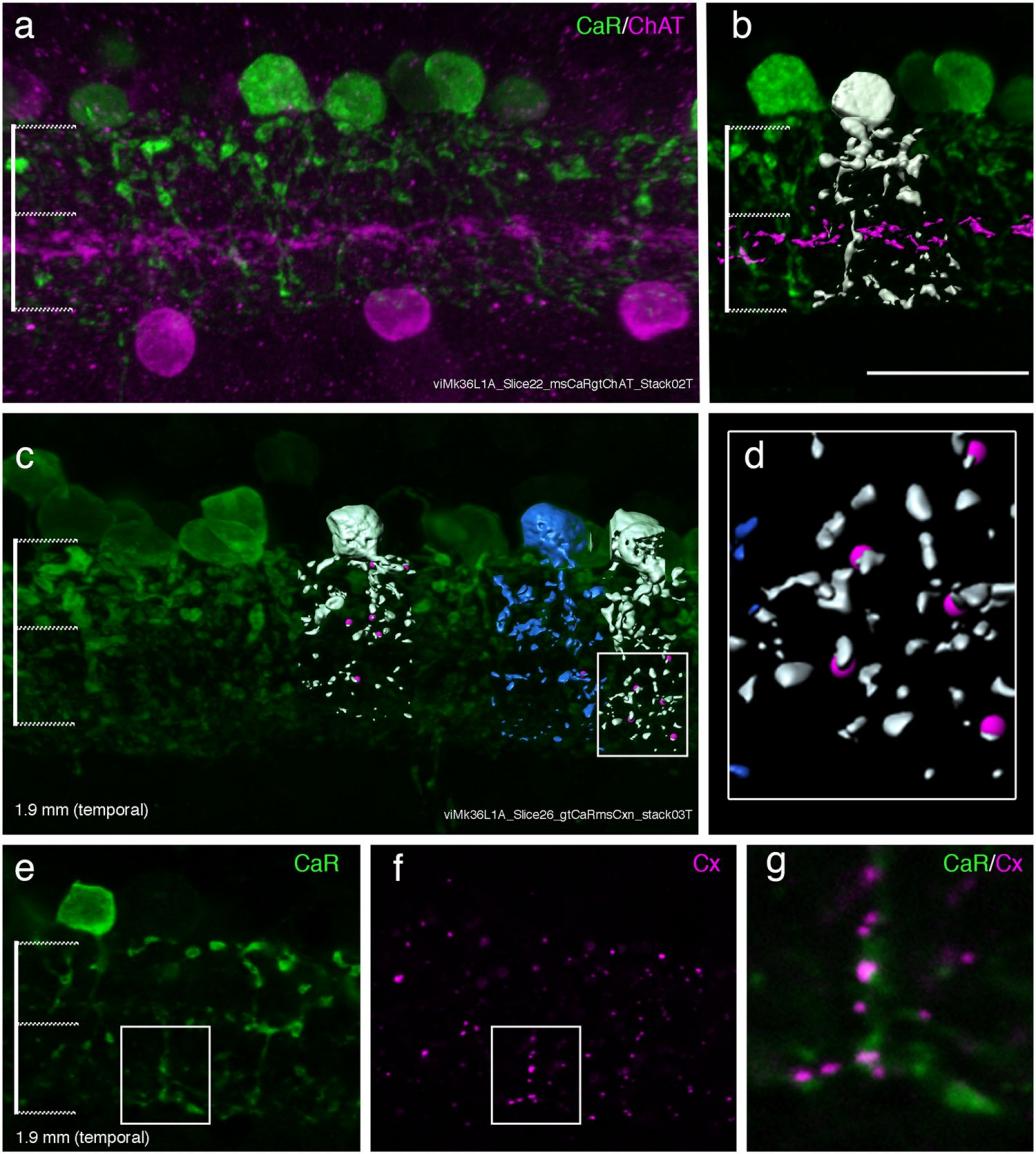
Gap junctions of AII cells. Confocal images of a vibratome section through central retina of macaque. (**a**) The section was processed with antibodies against calretinin (CaR) and antibodies against choline acetyltransferase (ChAT). (**b**) Shows a reconstructed AII cell from the section shown in a. The lines on the left indicate the OFF and ON sublamina. (**c**) Three-dimensional view of a section as processed with antibodies against calretinin (green) and connexin 36 (not shown). Three reconstructed AII cells are shown together with co-localized gap junctions (magenta). The box indicates the region shown in d. (**e,f**) Single confocal slice of a calretinin labelled AII cell (green) and the connexin immunoreactivity (magenta) in the same region. (**g**) Is the merged image of e and f. Scale bar shown in b = 20 µm (valid for a, b, c, e, f).

Connexin36 positive puncta associated with AII cells indicate sites of presumptive heterologous gap junctions established with ON cone bipolar cells as well as of homologous gap junctions with adjacent AII amacrine cells. We double labelled vertical sections with antibodies against calretinin and choline acetyltransferase to verify our distinction between the ON and the OFF sublamina (Fig. 6a,b). Adjacent sections were labelled for calretinin and connexin (Fig. 6c–g) to determine the connexin 36 positive puncta within 0.1 µm of the surface of AII dendrites. As can be seen in Fig. 6g, connexin immunoreactive puncta are closely associated with AII dendrites located in the ON sublamina.

**Figure 7:**
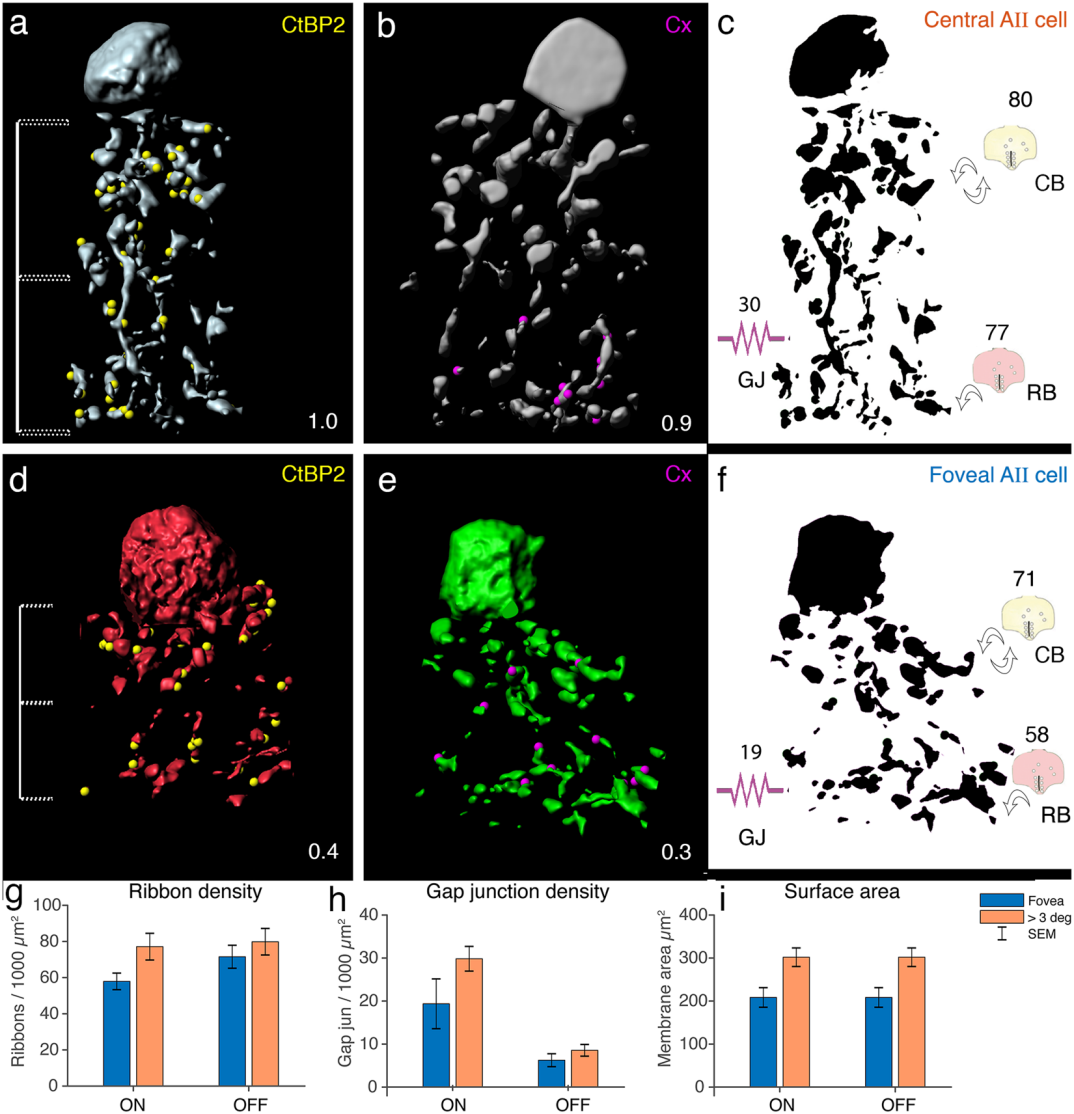
Comparison of AII cells in foveal and central retina. (**a,b,d,e**) Three dimensional reconstructions of AII cells from central and foveal retina together with synaptic ribbons (yellow) and gap junctions (magenta) summarizing our findings. Numbers in the bottom right represent eccentricities in mm. (**c,f**) Schematic drawings of the distribution of CtBP2 immunoreactive puncta (symbolized as ribbons in bipolar ending) and Cx36 immunoreactive puncta (symbolized as electrical resistor). The values represent the average number of immunoreactive puncta per 1000 µm^2^ dendritic surface area of reconstructed AII dendrites. (**g–i**) Histograms of ribbon density, gap junction density and surface area of reconstructed AII cells as function of retinal eccentricity and laminar position. CB, cone bipolar; RB, rod bipolar, GJ, gap junction.

Figure 7 summarizes the quantitative results. As expected, outside the fovea, the dendritic surface area of AII amacrine cells increases (on average by 25%, Fig. 7i, p = 0.02, Wilcoxon non-parametric rank-sum test) and the density of associated CtBP2 positive puncta also increases (by 23%, Fig. 7g, p = 0.03, Wilcoxon non-parametric rank-sum test). In foveal AII cells the number of ribbons assumed to represent rod bipolar cell input (58 ribbons/1000 µm^2^ surface area) is lower than the number of ribbons associated with cone bipolar cells (71 ribbons/1000 µm^2^). In contrast, outside of the fovea, the number of ribbons associated with AII cells is comparable for the OFF (80 ribbons/1000 µm^2^) and the ON (77 ribbons/1000 µm^2^) sublamina. Figure 7g also shows that the increase in associated CtBP2 puncta is greater for dendrites in the ON sublamina (+33%, p = 0.02, Wilcoxon non-parametric rank-sum test) than for dendrites in the OFF sublamina (+11%, p = 0.27, Wilcoxon non-parametric rank-sum test). This differential increase may be a consequence of increased contacts from rod bipolar cells outside the fovea.

On average foveal cells had a lower density of gap junctions (n = 19 per /1000 µm^2^) than AII cells outside of the fovea (n = 30; p = 0.02, Wilcoxon non-parametric rank-sum test, Fig. 7h). For both foveal and extra foveal AII amacrine cells, the density of connexin positive puncta associated with their dendrites is over 3 times higher in the ON sublamina compared to the OFF sublamina (Fig. 7h, fovea p = 0.02, extra-fovea p < 0.01, Wilcoxon non-parametric rank-sum paired test).

## Discussion

We have here provided anatomical evidence that AII amacrine cells contribute to ON-OFF crossover inhibition in the fovea, where rods are scarce or absent. Our study confirms that in human and macaque retina, most calretinin positive cells in the fovea are glycinergic (non-GABAergic) amacrine cells with the typical bistratified morphology of AII amacrine cells^17–21^. In common with other foveal neuron populations, the foveal AII cells are smaller than their counterparts outside the fovea, but they are nevertheless clearly identifiable. We combined calretinin staining with immunolabelling of synaptic ribbons using antibodies against CtBP2 or of gap junctions using Cx36 antibodies to compare the connectivity of foveal AII cells with that typical of more peripheral eccentricities. We found that with increasing eccentricity, AII amacrine cells develop a more distinct bistratified morphology, have larger dendritic arbours, and engage with higher numbers of ribbon synapses and gap junctions. Densities of both ribbon synapses and gap junctions increase with eccentricity, but the increase is more pronounced in the ON sublamina than in the OFF sublamina.

For both foveal and extra-foveal AII amacrine cells, the number of associated ribbons is comparable in the ON and OFF sublamina of the inner plexiform layer, suggesting that contacts with both ON and OFF bipolar cells are present in these cells. Consistently, EM and LM studies of macaque retina have shown synaptic input from AII amacrine cells to OFF bipolar cells^31,34^. These studies also suggested that the majority of this input is onto OFF (flat) midget bipolar cells with some input going to diffuse bipolar cells. Similarly, studies of rat^35^ and mouse retinas^32^ have shown that the AII amacrine cells preferentially contact one type of OFF bipolar cell. Full reconstructions of AII cells, however, will be needed to understand the AII to cone bipolar circuitry in primate fovea.

Connections from AII cells to OFF cone bipolar cells are known to be reciprocal and have been observed in electron microscopic studies of rabbit^5^, rodent^32,36^ and primate retinas^17,31^. The bipolar cell types involved with these reciprocal synapses in primates include the flat midget bipolar and the calbindin positive diffuse DB3a cell^31,34,37^ but, as said above, full reconstructions of AII cells will be needed to clarify this circuitry in primate fovea. Synaptic transmission between OFF cone bipolar cells and AIIs has also been demonstrated electrophysiologically^38^. However, the relevance of this input, which is opposite to the sign of synaptically-mediated ON inputs from rod bipolar cells (in scotopic vision); and of gap-junction mediated ON inputs from ON bipolar cells (in photopic vision) remains unclear^7^. This interesting puzzle notwithstanding, the presence of inputs from OFF bipolar cells underscores the involvement of AII cells in cone circuitry, and the high densities of ribbons found in the OFF sublamina is consistent with the finding that multiple OFF cone bipolar types connect to AII cells^31,34,39^.

Cx36 gap junctions are known to comprise both homologous connections with other AII amacrine cells as well as heterologous connections with ON cone bipolar cells^30,40,41^. Consistently, the number of Cx36 positive puncta found in the ON sublamina was substantially higher than in the OFF sublamina. Since AII cells are not expected to make gap junctions in the OFF sublamina^42^ we suspect these puncta are false positives deriving from gap junctions between cone bipolar cell axon terminals^39,43–45^, which arborize near AII lobular appendages. In sum, we found that the fundamental morphology and synaptic architecture are maintained for AII amacrine cells at foveal and extra-foveal locations: the contribution of rod bipolar cells outside the fovea appears superimposed on the existing, cone-driven circuitry of AII amacrine cells without major changes.

Our study has established new details of primate retinal wiring, but more broadly our findings are of relevance to evolutionary aspects of retinal circuitry^5,38^. It is known that the fundamental architecture of the retina has been dictated by cones, which are phylogenetically more ancient than rods^46,47^. Primordial vertebrates possessed only cone-like photoreceptors, and parallel processing of cone signals was likely established by subsets of cone bipolar and amacrine cells at the time when rods appeared^46^. Evolution of rod photoreceptors in turn supported the emergence of rod bipolar cells, which “piggy-backed” onto AII amacrine cells to access pre-existing retinal circuits for form, colour, and motion processing. By examining AII amacrine cells in the foveal, rod-free area, we may have emulated the primordial architecture of these neurons and demonstrated their reciprocal connections with ON and OFF cone bipolar cells. An additional (but not mutually exclusive) scenario is that ON-OFF crossover inhibition evolved together with rod bipolar cells, and was retained because of its functional value, even when rods disappeared from the area that eventually became the fovea in diurnal primates. In either scenario, and similarly to what was observed in peripheral primate retina (and in retinas of many non-primate species), in the foveal AII/cone bipolar network the ON system can exert a crossover inhibition on the OFF channel.

Because it is a small-field amacrine cell involved in local, inhibitory circuits with cone bipolar cells, foveal AII cells could help to shape the spatial and temporal properties of cone-driven signals. It appears that presence of rod-driven circuitry outside the fovea does not replace or degrade the connections with cone bipolar cells but creates a more complex functional architecture which is shared by scotopic and photopic pathways.

## Materials and Methods

### Tissue collection and preparation

Two retinas from one male and one female adult macaque monkeys (*Macaca fascicularis*) were obtained after unrelated electrophysiological experiments approved by the Animal Ethics Committee of The University of Melbourne (Ethics ID 0701761.4). All procedures conformed to the provisions of the Australian National Health and Medical Research Council code of practice for the care and use of animals. At the end of the electrophysiological experiments the animals were overdosed with a lethal intravenous dose of pentobarbitone sodium (5 ml of Nembutal, 60 mg/ml; Merial Australia) and transcardially perfused with 0.1 M phosphate buffered saline (PBS) followed by 4% paraformaldehyde in PBS. Subsequently, the eyes were enucleated, cut open, and the posterior eyecup was immersion fixed for 30 min or 60 min in the same fixative.

Human donor eyes (with corneas removed) with no known history of posterior eye disease were received with informed consent from the Lions NSW Eye Bank and Australian Ocular Biobank and with ethical approval by The University of Sydney Human Research Ethics committee (HREC# 2012/2833). The protocols adhered to the Declaration of Helsinki. The eyes (two females, aged 44 years, one male, aged 53 years) were received within 3.3, 4.3 and 9.7 hours after death. The posterior eye cups were immersion fixed in 2% paraformaldehyde in 0.1 M phosphate buffer for 12 to 63 hours. After rinses in 0.1 M PB, and the retina was dissected.

Quadrants of monkey and human retinas were immersed in 30% sucrose overnight, quick frozen using liquid nitrogen and stored at −80 °C until use. Retinal pieces of about 3 mm by 4 mm centred on the fovea were cut out and embedded in low melting Agarose. The pieces were then sectioned along the horizontal meridian at 100 µm thickness using a Vibratome (VT 1200 S, Leica Microsystems, Nußloch, Germany). The foveal region of one macaque retina was sectioned at 14 µm thickness using a cryostat (CM3050S, Leica).

### Immunohistochemistry

Primary antibodies used in the present study are summarized in Table 1. Vibratome sections were processed free floating, cryostat sections were processed mounted on slides. Sections were preincubated in PBS containing 5% normal donkey serum and 0.5% Triton X-100 for 1 hour or overnight. Subsequently, the sections were incubated with two or three primary antibodies for 7 days at 4 °C (or overnight at room temperature for cryostat sections) diluted in PBS containing 3% normal donkey serum and 0.5% Triton X 100 and 0.1% sodium azide. Secondary antibodies (made in donkey) were obtained from Jackson ImmunoResearch (West Grove PA). They were diluted in PBS containing 3% donkey serum and 0.5% Triton X 100 and applied for about 16 hours at 4 °C. Retinal sections were mounted with Vectashield mounting medium (H-1000 Vector laboratories, Burlingame, CA). For vibratome sections we used secure seal spacers (Invitrogen) to prevent squashing of the sections.

**Table 1:** Antibodies.

Antibody name	Immunogen	Source, Catalogue number, RRID	Antibody type	Dilution
Calretinin	Amino acids 38–151 of rat calretinin	BD Biosciences, North Ryde, Australia, 610908, clone 34; RRID: AB_398225	Mouse, monoclonal	1:2000
Calretinin	Recombinant human calretinin	Swant, Marly, Switzerland, CG1 RRID: AB_10000342	Goat, polyclonal	1:5000
GAD-6	Affinity purified glutamic acid decarboxylase from rat brain	Developmental Studies Hybridoma Bank developed under the auspices of the NICHD and maintained by the University of Iowa, IA; RRID: AB_528264	Mouse, monoclonal supernatant	1:100
GAD-67	Recombinant GAD67 protein	Millipore Cat#MAB5406; RRID: AB_2278725	Mouse, monoclonal	1:8000
GlyT1	Synthetic peptide corresponding to the final 15 amino acids of the C-terminal region of glycine transporter 1 coupled to porcine thyroglobulin using formaldehyde	Gift from David Pow, University of Queensland, Australia; RRID: AB_2314597	Rabbit, polyclonal	1:5000
PKCα (clone MC5)	Purified bovine brain protein kinase C, epitope located within the amino acid sequence 296–317 at the hinge region (close to or at the trypsin cleavage site) of PKC	Sigma-Aldrich Cat# P5704, RRID: AB_477375	Mouse, monoclonal	1:1000
PKCα	Synthetic peptide corresponding to amino acids 659–672 from the C-terminal variable (V5) region of rat PKC α conjugated to KLH.	Sigma-Aldrich Cat# P4334, RRID: AB_477345	Rabbit, polyclonal	1:50,000
Rhodopsin (clone Rho 1D4)	Bovine rhodopsin, C-terminus, last 9 amino acids	Millipore Cat#MAB5356, RRID: AB_11215453	Mouse, monoclonal	1:200
S-antigen	Highly purified bovine S-antigen (rod arrestin)	Gift from Leo Peichl, Max Planck Institute for Brain Research, Frankfurt, Germany	Rabbit, polyclonal	1:1000

### Microscopy

Images were taken with a confocal microscope (LSM700, Carl Zeiss) equipped with 405, 488, and 555 nm lasers and using a 20× air objective (Plan Apochromat, 20×/0.8, #420650-9901-000) or a 40x water immersion objective (C-Apochromat, 40×/1.2, #421767-9970-000). Tiled image stacks of vibratome sections were taken at a resolution of 2048 by 2048 pixels and a step size of 0.89 µm. The tiled image stacks were subsequently stitched together using Zeiss ZEN black edition software. The contrast and brightness of the images was adjusted using Zen Black, Adobe Photoshop, or Imaris (Bitplane, Zurich, Switzerland) software.

### Analysis

Image stacks were analysed using Zeiss Zen Blue Edition Software for the soma counts. Imaris software was used for the ribbon and gap junction analysis. Briefly, individual AII cells were reconstructed using the surface area tool and the surface area of the two dendritic tiers located in the ON and OFF sublamina of the inner plexiform layer was measured. Separation of sublamina a from sublamina b was determined using morphological landmarks (position of bipolar cell endings or staining with antibodies against choline acetyltransferase to reveal the cholinergic bands). Subsequently, CtBP2 immunoreactive puncta (ribbons) and connexin 36 immunoreactive puncta (gap junctions) were detected using the detect spots tool. Next, we identified CtBP2 immunoreactive puncta within a distance of 0.3 µm of the surface and the connexin immunoreactive puncta within a distance of 0.1 µm of the reconstructed surface. We then calculated the immunoreactive puncta per 1000 µm^2^ surface area. The eccentricity of individual AII cells was calculated, taking into account the distance (if any) of the source Vibratome section from the horizontal meridian.

### Statistical analysis

Statistical comparisons were made using the Matlab (Mathworks, Natick, NJ) statistics toolbox. Percentage variances are expressed as standard deviation (SD); density/area variances are expressed as standard error of mean (SEM). Because normality of underlying distributions cannot be assumed, sample comparisons used Wilcoxon’s non-parametric rank-sum test (two-sided). All comparisons are based on >20 samples therefore small sample size corrections were not applied. Exact p-values are reported for values above 0.01.

## Data Availability

The datasets generated and analysed as part of the current study are available from the corresponding author upon request.
